# Bibliography

**Published:** 1897-05

**Authors:** 


### Bibliography.

TRANSACTIONS OF THE AMERICAN DENTAL ASSOCIATION AT THE THIRTY-SIXTH ANNUAL SESSION, held at Saratoga Springs, N. Y., commencing on the 4th of August, 1896. Philadelphia: S. S. White Dental Manufacturing Company, 1897.

This report is very satisfactory in one respect, at least, that it has appeared in good season and is a very creditable volume in general make up. The meeting at Saratoga was not all that it should have been, being especially weak upon the practical side of dentistry, but some of the papers are of special interest, especially so that on "Prosthesis Extraordinary," by Dr. E. A. Bogue, describing two operations by Dr. Michaels, of Paris, one on the restoration of the jaw to its original length, and the other on replacing the upper portion of the humerus, and both very successful, platinum and hard vulcanized rubber being used in both instances. This is certainly not only new in prosthetic dentistry, but reflects the highest honor on Dr. Michaels. It is worthy of special notice as showing what can be accomplished by an original mind coupled with mechanical skill.

The paper, by Dr. W. D. Richards, on "Artificial Pulps" is a somewhat original conception, and gives a very clear idea of the variety of forms assumed by pulp-canals, and enforces the statement of the essayist that this exhibit "would lead us to consider, after viewing what a tooth may contain, how far we may err in attempting to file pulp-canals perfectly, and how *immediate* root filling should be regarded."

The paper by Dr. L. P. Bethel, on "Lining Root-Canals" with nitrate of silver through the agency of the electric current (cataphoresis), is a paper of marked practical interest, and elicited much discussion for and against the views sought to be established.

In the same line may be classed Dr. Joseph W. Wassell's paper on "The Disinfection of Pulpless Teeth," and worthy of careful reading.

The paper by Dr. W. C. Barrett, on "The Orbicularis Oris and the Muscles of Expression," is one requiring special study, and this should be given it by all dentists who replace the natural organs with artificial dentures.

The reports and papers by Dr. J. S. Cassidy on "Materia Medica and Therapeutics," Dr. J. D. Patterson on "Physiology and Etiology," Dr. Thomas Fillebrown on "The Study of the Relation of the Frontal Sinus to the Antrum," Dr. H. L. Ambler on "Nodule on Apex of Root," Dr. Louis Ottofy on "Dental Education, Literature, and Nomenclature," and Dr. S. H. Guilford, with an exhaustive article upon the latter subject, all show careful preparation, and furnish valuable material for study and reference.

It is to be hoped that at the next meeting at Old Point Comfort, in August, 1897, the sections having in charge the practical side of dentistry will make an equally good presentation.

Annual of the Universal Medical Sciences and Analytical Index. A Yearly Report of the Progress of the General Sanitary Sciences throughout the World. Edited by Charles E. Sajous, M.D., Paris, and seventy associate editors, assisted by over two hundred corresponding editors, collaborators, and correspondents. Illustrated with chromo-lithographs, engravings, and maps. Philadelphia, New York, Chicago: The F. A. Davis Company, 1896.

This invaluable production of the incessant labor of Dr. Sajous and his collaborators is again presented to the medical public. These five large volumes comprise the work of the world in medical science during the year 1896.

Many of the abstracts are necessarily brief, but others are of reasonable length, and this must be regarded as a wise change, for it is the fault of all such compendiums that they frequently, by too great brevity, distort the meaning of writers. This has been appreciated by the editor, for he says, "To bring the usefulness of the Annual to its highest standard it was deemed advisable to increase the length of the abstracts so as to fully convey an author's meaning."

The editor further says in his preface, "While one-half a million words added to the Annual greatly enhance the value of each subject treated, the increase will naturally impose greater labor upon the physician who wishes to acquaint himself with the progress of the medical sciences as a whole."

To meet this difficulty a new department has been arranged, which is described as follows: "The Analytical Index and Cyclopædia of Treatment gives a summary of every practical article quoted in the Annual proper, and of all the criticisms introduced by associate editors. . . . The arrangement of this material is peculiar, a counterpart, as it were, of an international medical congress in brief, each subject being divided into sections." This arrangement of the editor to facilitate examination cannot be too highly commended. Covering, as it does, about three hundred and fifty pages in good, clear type, it will enable the busy practitioner to readily cover the entire field of the year's work in a short time.

The improvements in this series of volumes over that of previous issues may be found in "headings and side-headings, presented in large, black letters. All prescriptions are written out in full, and in the usual form. . . . All therapeutic subjects have been collected in the fifth volume. . . . Increase in the number of colored plates. . . . "

It has been a matter of regret, expressed in previous notices, that dentistry finds no place anywhere in these volumes. It is recognized that this has not been the fault of the editor, but exactly why this has not been done the writer has not been informed. It is rather late in the nineteenth century to permit old prejudices to enter into the production of this work, and it is not believed they have had any force in the omission, but whether or not the time has arrived when this branch of medicine should be made a part of the general healing art, for it is certainly of equal importance, and, in the writer's estimation, not in the least inferior to any of the so-called specialties of medicine.

The same careful work is evidenced throughout these five volumes as in those previously issued, but the immense amount of labor given to their preparation cannot be appreciated by those not connected with similar work.

It is understood that Dr. Sajous, the editor, will remove to Philadelphia, and again place the editorial department of the Annual where it was originally established.

Clinique Dentaire et Dentisterie Opératoire. Par Ch. Godon, Directeur de l'École dentaire de Paris. Avec 62 figures intercalées dans le texte. Librairie J. B. Baillière et Fils, Paris, 1897.

This small volume is one of a series of books in the form of manuals, several of which have heretofore been noticed. This book is principally confined to operative dentistry, with such allusions to pathological conditions as seem necessary.

It is impossible in so small a work to do justice to the subjects treated, hence a book of this kind must be judged by another standard than ordinary productions. Its value consists in its brevity, a virtue especially appreciated by the overtaxed student. As it is a refresher of memory it can be recommended to French students who, it may be presumed, require such aids as well as their American brethren. Not a few on this side of the water depend largely upon similar manuals for a large proportion of knowledge obtained.

Catching's Compendium of Practical Dentistry for 1896. B. H. Catching, D.D.S., editor and publisher. Atlanta, Ga., 1897.

We are again presented with this valuable *résumé* of the dental work of the year 1896, published at an early period in the year 1897.

This journal from year to year, as this important book of reference has appeared, has given it a cordial recognition. There has been a lingering fear that the able editor and publisher, Dr. Catching, would find this task too burdensome and perhaps too unremunerative to continue; but evidently the end is not yet, if it is ever to come, for here is a volume of eight hundred and seventy-eight pages, neatly printed on good paper, and giving in condensed form the valuable matter of the year. When necessary the articles are given almost entire; especially is this the case in the scientific papers. This is a wise procedure, as frequently attempts at abstracting these papers make the matter almost valueless.

Dr. Catching has succeeded, as heretofore, in condensing the substance of many articles, but few, it is presumed, will ever think of the great labor required to sift this from the mass of verbiage presented in the dental periodicals.

The product of each year, when thus brought together, does not make a very remarkable exhibit; in fact, original investigation is not a marked characteristic of dentistry in general, but it will be observed, both at home and abroad, that there is always a small contingent at work seeking to solve its remaining problems. As an historical record and a work of reference, this book has been, and will continue to be, of special value, and to the overworked dentist who cannot keep up with the monthly journals, but who still desires to have in his library the yearly work of his profession, this book is essential, and the general practitioner, who finds time for everything, needs it to refresh his memory in matters read but lost sight of in the busy whirl of active life.

